# Galactose-1-phosphate uridyltransferase (GalT), an *in vivo*-induced antigen of *Actinobacillus pleuropneumoniae* serovar 5b strain L20, provided immunoprotection against serovar 1 strain MS71

**DOI:** 10.1371/journal.pone.0198207

**Published:** 2018-06-01

**Authors:** Fei Zhang, Qin Zhao, Keji Quan, Zhuang Zhu, Yusheng Yang, Xintian Wen, Yung-Fu Chang, Xiaobo Huang, Rui Wu, Yiping Wen, Qigui Yan, Yong Huang, Xiaoping Ma, Xinfeng Han, Sanjie Cao

**Affiliations:** 1 Research Center of Swine Disease, College of Veterinary Medicine, Sichuan Agricultural University, Chengdu, China; 2 National Teaching and Experimental Center of Animal, Sichuan Agricultural University, Chengdu, China; 3 Sichuan Science-observation Experimental Station of Veterinary Drugs and Veterinary Diagnostic Technology, Ministry of Agriculture, Chengdu, China; 4 Department of Population Medicine and Diagnostic Sciences, College of Veterinary Medicine, Cornell University, Ithaca, NY, United States of America; Instituto Butantan, BRAZIL

## Abstract

GALT is an important antigen of *Actinobacillus pleuropneumoniae* (APP), which was shown to provide partial protection against APP infection in a previous study in our lab. The main purpose of the present study is to investigate GALT induced cross-protection between different APP serotypes and elucidate key mechanisms of the immune response to GALT antigenic stimulation. Bioinformatic analysis demonstrated that *gal*T is a highly conserved gene in APP, widely distributed across multiple pathogenic strains. Homologies between any two strains ranges from 78.9% to 100% regarding the *gal*T locus. Indirect enzyme-linked immunosorbent assay (ELISA) confirmed that GALT specific antibodies could not be induced by inactivated APP L20 or MS71 whole cell bacterin preparations. A recombinant fusion GALT protein derived from APP L20, however has proven to be an effective cross-protective antigen against APP sevorar 1 MS71 (50%, 4/8) and APP sevorar 5b L20 (75%, 6/8). Histopathological examinations have confirmed that recombinant GALT vaccinated animals showed less severe pathological signs in lung tissues than negative controls after APP challenge. Immunohistochemical (IHC) analysis indicated that the infiltration of neutrophils in the negative group is significantly increased compared with that in the normal control (P<0.001) and that in surviving animals is decreased compared to the negative group. Anti-GALT antibodies were shown to mediate phagocytosis of neutrophils. After interaction with anti-GALT antibodies, survival rate of APP challenged vaccinated animals was significantly reduced (P<0.001). This study demonstrated that GALT is an effective cross-protective antigen, which could be used as a potential vaccine candidate against multiple APP serotypes.

## Introduction

Pig contagious pleuropneumonia (PCP) is one of the primary diseases of respiratory system in swine, causing swine cellulose pleurisy and pneumonia. APP is the etiologic agent of this disease. The murine model for APP respiratory tract infection has been experimentally developed to evaluate the efficacy of vaccines [1]. According to previous studies, this model is a far less laborious and resource intensive than the standard porcine model[2, 3]. 15 serovars have been reported over the past several decades, and a new serovar 16 was reported in 2017[4, 5]. Traditionally, protection obtained from immunization has been serovar-specific; vaccines have typically lacked cross protective ability between different serovars[6]. As a result, vaccines had to be made from prevalent serovars within a given region, and have proven to be of little use in other areas where different serovars predominate [6]. Multivalent inactivated vaccines against APP have incorporated antigens from many serovars, and thus are more useful across multiple regions. Multivalent vaccines were shown to provide limited cross protection and partially reduced the incidence of the disease [7, 8].

To improve the performance of first generation inactivated vaccines, other targets have been studied recently [9, 10]. The most promising vaccine candidates are cross protective antigens; typically these are immunogenic markers of pathogens highly conserved across multiple serovars [11]. Virulence factors of APP like Apx toxins, lipopolysaccharide, and capsular polysaccharides have been studied as cross-protective vaccine candidates [10, 12, 13]. ApxI, ApxII and ApxIII have proven to provide cross protection against different APP serovars [7, 10, 12]. ApxIV is another Apx toxin that possesses strong antigenic properties, the gene of which is conserved across all 16 known serovars of APP. A recombinant rApxIVN has been shown to induce a high level of antibodies and confers strong protection against challenges with different APP serovars[10]. Antigens that are conserved across many serovars of a given pathogen could be considered as potential components in cross protective vaccine development. Most microbial pathogens are isolated and cultured on artificial media (*in vitro*) in a clinical setting[14, 15]. Many virulence genes, including those that code for highly antigenic virulence factors, are inducible and expressed only *in vivo* [16]. Additionally, *in vivo* study of pathogens in hosts provides an opportunity to investigate humoral and cellular immune responses to infection. Many experimental methods have been developed for studying and targeting *in vivo*-induced genes such as signature-tagged mutagenesis (STM), in vivo expression technology (IVET), selective capture of transcribed sequences(SCOTS), all of which have been widely used to screen antigens of microbial pathogens [17–19]. These technologies have proven to be powerful tools in efforts in the development of cross reactive vaccines [20].

*In vivo*-induced (IVI) antigens play an important role in virulence to, or colonization of the host [21–27]. IVI proteins are often both highly antigenic and highly conserved across multiple serovars of a given pathogen, making them ideal candidates for cross-protective vaccines[28–31]. Galactose-1-phosphate uridyltransferase (GALT), belongs to *gal* gene cluster, the products of which are enzymes involved in metabolism of galactose[32].These genes are involved in the LPS core biosynthesis and therefore key virulence factors[33, 34]. GALT is an APP *in vivo*-induced antigen and our previous study has proved that this protein is highly immunogenic and provides partial immune protection against APP challenge [35, 36].

In the present study, cross-protection and the mechanism of immune response of GALT were investigated in mice. GALT induced cross-protection against APP serovar 1 or serovar 5b challenge. Anti-GALT serum can mediate neutrophil killing function. GALT was an effective cross-protective antigen and was a potential vaccine candidate for prevention of PCP.

## Material and methods

### Ethical approval and animal care

All animal experiments performed in this study were approved by the Institutional Animal Care and Use Committee of Sichuan Agricultural University (Approval Number BK2014-047). Six to eight week female BALB/c mice (18-22g) used in the present study were purchased from Chengdu Dossy Experimental Animal Co., Ltd. The animals were fed with food and water *ad libitum*. Meanwhile, wood wool was provided as nesting material. All animals kept were checked daily. Mice were euthanized immediately at the end of animal experiment, or when host animals displayed the onset of severe clinical signs of APP infection. Every effort was made to minimize animal suffering.

### Bacterial strains, media and growth condition

Trypticase Soy Agar (TSA) or Trypicase Soy Broth (TSB) with 5% (v/v) fetal calf serum was used for APP strain cultivation, and nicotinamide adenine dinucleotide (NAD; 15 mg/mL) was added as growth factor. APP reference strains were purchased from the China Institute of Veterinary Drug Control (Beijing, China); some APP isolated strains were stored and maintained in our lab (Table 1). GALT expression strain *E*. *coli* BL21 (pET-*gal*T) used in this study was transformed with the recombinant plasmid constructed in our previous study[36]. *E*. *coli* was cultured in Luria–Bertani (LB) medium with kanamycin (50 mg/mL). Both APP strains and *E*. *coli* strain were cultured at 37°C, 220 rpm.

**Table 1:** Bacterial strains used in this study.

Bacterial strain	Serotype	References
Shope 4074 (ATCC27088)	1	[65]
S1536 (ATCC27089)	2	[65]
S1421 (ATCC27090)	3	[65]
M62 (ATCC33378)	4	[65]
L20	5b	[65]
K17 (ATCC33377)	5a	[65]
Femo SCI-A(ATCC33590)	6	[66]
WF83	7	[65]
F384	8	[66]
F60	9	[66]
D13039	10	[65]
MS71	1	[65]
MS33	1	[65]
MS52	1	[65]
MS53	1	[65]
MS54	1	[65]
GA16	7	[65]

### Bioinformatics analysis

*Apx*IVA genes were amplified by PCR from APP reference and isolated strains using specific primers for species identification (Table 2). The sequences of *gal*T from L20, JL03 and AP76 were obtained for NCBI and the conserved sequences were analyzed by DNASTAR software, which was used to design primers. Primers of *gal*T were designed using primer premier 5 software. *Gal*T genes of all of the strains were amplified by PCR and sequenced (Qingke Biotech, Chengdu, China)(S1 File). The nucleotide sequences of *gal*T of APP and other bacteria of the genus *Actinobacillus* were analyzed using L20 *gal*T sequence via BLAST software and saved for further use. Multiple sequence alignments and the identities between each pair of nucleotide sequences were calculated by the Jotun Hein method using the MegAlign program[37]. ClustalX1.83 and MEGA5.05 were used to construct a phylogenetic tree of *gal*T genes of the studied strains. The phylogenetic tree was set as 1, 000 bootstrap replicates and the Neighbor-Joining method was used[38].

**Table 2:** PCR primer sequences used in this study.

Gene	Upstream sequence	Downstream sequence	Size(bp)	References
*Gal*T	5’-CACCCTCATCGTCGTTTT-3’	5’-TCCGCCATCATCTCGTAG-3’	949	This study
*Apx*IVA	5’-GACGTAACTCGGTGATTGAT-3’	5’-GAATTCACCTGAGTGCTCACCACC-3’	388	[66]

### Expression and purification of recombinant protein

*E*. *coli* BL21 (pET-*gal*T) was cultured overnight. The culture production was added in new LB media in 1:100 for protein expression. IPTG (1 mmol/L) was added when the OD600 of the culture reached OD0.6. After induction for another 4 hours, bacteria were collected by centrifugation at 12000 g, for 10 min. Prepacked low-pressure chromatographic cartridges (Bio-Rad, USA) was used in this study and solution was prepared according to its instruction manual. Bacteria were suspended in 40 mL 1×binding buffer (Bio-Rad, USA) and then treated with ultrasonication in 4°C. Lysate was centrifuged 12000 g for 10 min and the supernatant was collected for His tagged fusion protein purification. GALT His-tagged fusion protein was purified by Ni-affinity chromatography. The purified recombinant protein was stored at -80°C.

### Preparation of polyclonal antibody

Six-eight week (18-22g) BALB/c mice were randomly divided into four groups for preparation of polyclonal antibody. These groups were vaccinated subcutaneously with phosphate buffer (PBS) plus adjuvant, formalin-inactivated APP1(1×10^8^CFU/mice) plus adjuvant, formalin-inactivated APP 5b(1×10^8^CFU/mice) plus adjuvant and GALT protein (50 μg/mice) plus adjuvant, which were denoted as negative control group, APP1 group, APP5b group and GALT group, respectively. Freund's Complete Adjuvant was used in the initial immunization and a booster immunization was executed two weeks later with Freund's Incomplete Adjuvant. Four weeks post initial immunization, sera in different groups were prepared and sub-packed, and then stored in 80°C for subsequent protocols.

#### Vaccination and evaluation of immune protection

Six to eight week female BALB/c mice (18-22g) were randomly divided into 4 groups (eight mice per group) (Table 3). Two groups were immunized subcutaneously with GALT protein (50μg/mice) plus adjuvant and the other two groups as negative control, which was immunized subcutaneously with PBS plus adjuvant. The normal control was the group of mice not challenged with serotype 1 or 5b APP. Negative control group consisted of mice given PBS plus adjuvant only. Freund's Complete Adjuvant and Freund's Incomplete Adjuvant were used in the initial and booster immunizations. Boosting was carried out after initial immunization with a 2-week interval. Blank serum and test serum samples of two weeks post vaccination and booster were collected for detection of IgG. LD_50_ of APP1 and APP 5b was determined using a Reed–Muench method to obtain a standard challenge dose[39]. Two weeks post booster, PBS group and GALT protein group were challenged with a lethal dose APP1 or APP 5b, respectively. Seven days of continuous observation followed; surviving animals were noted.

**Table 3:** Details of animal immunization and challenge assay.

Group	Number of animals	immunization	Challenge Day28	Number of deceased mice							Survival rate
				Day1	Day2	Day3	Day4	Day5	Day6	Day7	
1	8	GALT(50μg/mouse)	APP5b L20 (3.24×10^8^ CFU)	1	1	0	0	0	0	0	6/8
2	8	GALT(50μg/mouse)	APP1 MS71 (3.10×10^7^ CFU)	2	2	0	0	0	0	0	4/8
3	8	PBS plus adjuvant	APP5b L20 (3.24×10^8^ CFU)	6	2	0	0	0	0	0	0/8
4	8	PBS plus adjuvant	APP1 MS71 (3.10×10^7^ CFU)	5	3	0	0	0	0	0	0/8

Note: CFU: colony forming unit.

#### Indirect enzyme-linked immunosorbent assay (ELISA)

Serum from immunized mice was examined by ELISA for IgG levels. In brief, 96-well plates were coated with 200 ng/100 μl (per well) of purified GALT recombinant protein diluted in 0.02 M carbonate–bicarbonate buffer (pH 9.6) overnight at 4°C. Wells were washed thrice with PBST, then blocked with 3% (w/v) bovine serum albumin (BSA) in PBST for 1.5 h at 37°C. The plates were washed three times and incubated with 100 μl sera diluted in 1:100 for 1 h at 37°C. Blank serum, serum from 2 weeks post initial and boosting immunization in negative group, APP1 group, APP5 group and GALT group were diluted in 1:100 using serum diluent and added to the wells. After three rinses, goat anti-mouse HRP–IgG (Bioss, China), diluted 1:5000 in PBS, was used as the secondary antibody and incubated for 1 h at 37°C. Plates were washed three times with PBST; TMB was added and plates incubated for 10 min in the dark at RT. The reaction was stopped with 2 M H_2_SO4. The absorbance of each well was read at a wavelength of 450 nm using an ELISA reader (Bio-Rad, USA).

#### Histopathology

Lungs of animals from different groups were isolated and were fixed by immersion in 10% neutral formalin. Immersed tissues were sectioned at 5mm thickness, and HE-stained for evaluation of histopathology using an Olympus DP71 microscope(Olympus, Japan). The galT immunized group and the non-immunized group challenged with APP 1 and APP 5b were analyzed. Lung tissues from healthy mice served as the normal control.

#### Immunohistochemistry analysis

A standard immunohistochemical (IHC) method was conducted to analyze neutrophil and macrophage infiltration in tissues from different groups[36]. IHC staining with monoclonal antibodies (MAbs) was carried out on paraffin wax sections obtained from different mouse lung sections. Two MAbs were used: rabbit anti-mouse CD68 for macrophages and rabbit anti-mouse myeloperoxidase (MPO) for neutrophils. Sections were treated as described. The stained IHC sections were examined in 200×magnification and photos taken. IHC photos were analyzed by Image-Pro Plus 6.0 software (Media Cybernetics, Inc., Rockville, MD, USA). Integrated optical density (IOD) of each photo was determined as positive index. The GALT immunized group and negative group challenged with APP 1 and APP 5b were analyzed for neutrophils and macrophages infiltration. Lung tissues from healthy mice were treated as normal control. The IHC results were analyzed using IHC Mean Density analysis method by Image-pro plus 6.0, which was introduced as follows: For each section, 3 fields at 100x magnification were selected randomly and photographed.

#### Isolation of mice bone marrow neutrophils

Mice bone marrow neutrophils were isolated using mouse bone marrow neutrophil isolation kit (Haoyang Biological, Tianjin, China). Mice femurs were isolated using sterile technique and bone marrow fluid was washed with cleanout fluid (anticoagulant was added) using a sterile syringe. The bone marrow suspension was centrifuged in 450 g for 15 min and the supernatant was discarded carefully. Cells were suspended with erythrocyte sedimentation liquid and cell density was adjusted to 2×10^8^ to 1 × 10^9^/ml. 3 mL of undiluted separating medium and 1.5 mL of 80% separating medium added(separating medium 1: sample diluent = 4:1) successively into a 15 mL centrifuge tube. Bone marrow suspension was added into the 15 mL centrifuge tube carefully and centrifuged at 800g for 20 min. After centrifugation, the white neutrophil layer was extracted. Five-fold volume cleanout fluid was added into the tube containing neutrophils and the tube was centrifuged in 400g for 10 min. The supernatant was discarded and erythrocytes were removed using erythrocyte lysis buffer.

#### Neutrophil killing assays

Neutrophils are of great importance in the process of interaction between bacteria and host. Mouse Neutrophil kill experiments were carried out to determine whether anti-GALT sera possessed opsonophagocytosis effect. The assay was carried out based on one used in a previous study with slight modification[40]. APP 5b cultures in logarithmic growth phase were washed 3 times with sterilized PBS, then suspended in RPMI-1640 culture and stored at 4°C. Neutrophils were suspended in RPMI-1640. Neutrophil suspension at a density of 1.0×10^6^ cells/mL was added to an equal volume (50 μL) of 1×105 CFU/mL APP 5b. Anti-GALT serum, blank control serum and negative serum (25μL) was mixed with cells to a 20% final concentration. Bacteria cultured alone were used as controls. After incubation for 1 hour at 37°C, the culture was diluted in sterile PBS and spread on TSA for colony-counts. The percentage of bacteria surviving opsonophagocytic killing was calculated as (CFU with neutrophils/CFU without neutrophils)×100%.

#### Statistical analyses

All data analyses were performed by SPSS 19.0 software using Student’s t-test for the comparison of the differences between different groups. P-values of<0.05 were considered as statistically different and were represented with asterisk. P-values of<0.001 were represented with two asterisks. NS denotes not significant.

## Results

### *Apx*IVA and *gal*T genes are widely distributed across APP serovars

**Fig 1:**
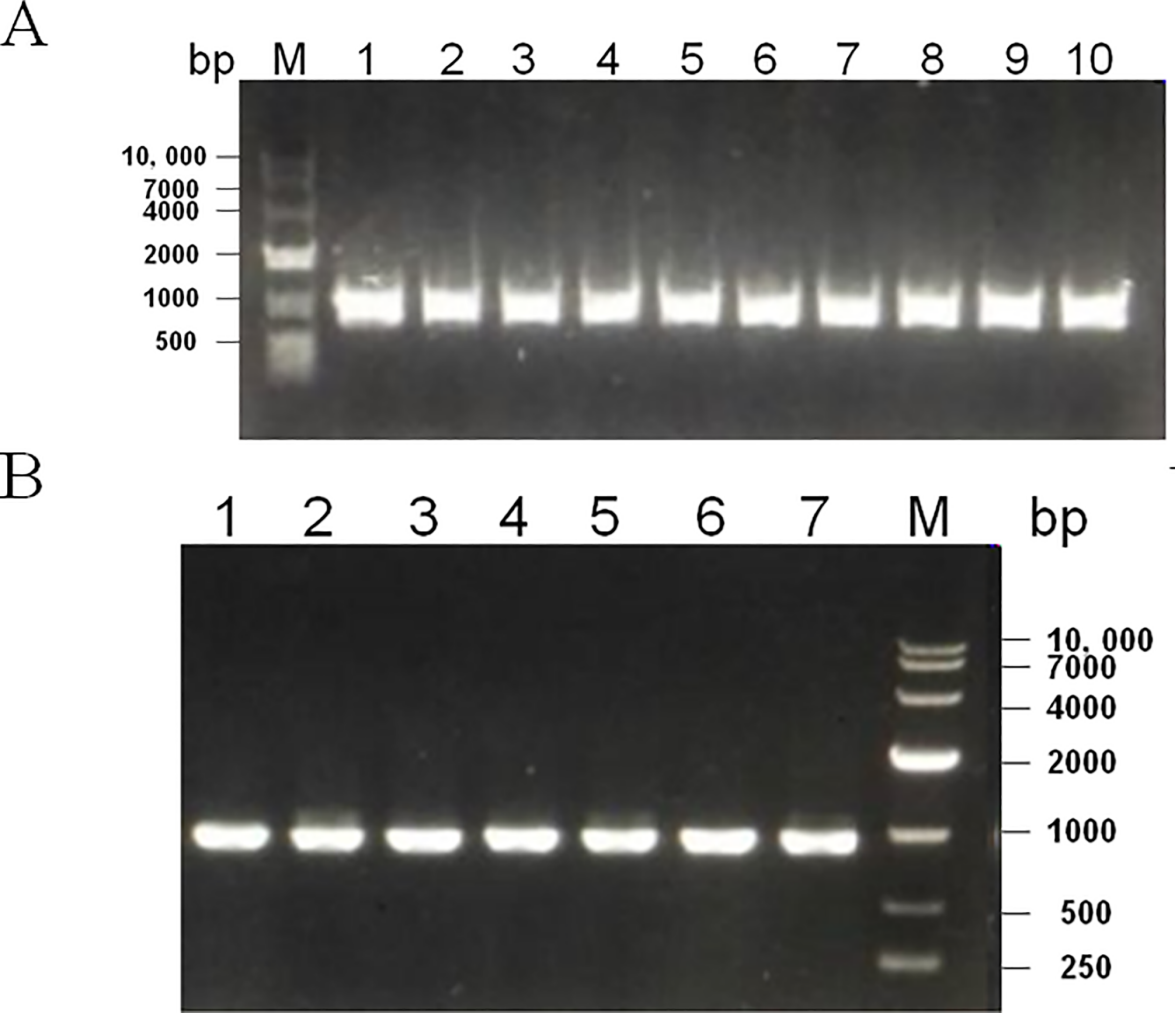
Detection of *Gal*T gene in various App strains by PCR. *galT* gene was amplified by PCR and analyzed using 1% agarose gel. (A)Lane1-10:Shope4074, S1536, S1421, M62, K17, L20, Femo SCI-A, WF-83, F384, and F60. (B)Lane1-6: D13039, GA16, MS33, MS52, MS53, MS54, MS71.

*Apx*IVA genes were amplified by PCR from all APP strains using specific primers (Data not shown). Also, using specific primers of *gal*T, *gal*T genes were amplified by PCR from all the APP strains used in this study. A fragment about 949 bp was amplified from APP strain after agarose gel electrophoresis(Fig 1).

### *Gal*T gene is highly conserved

**Fig 2:**
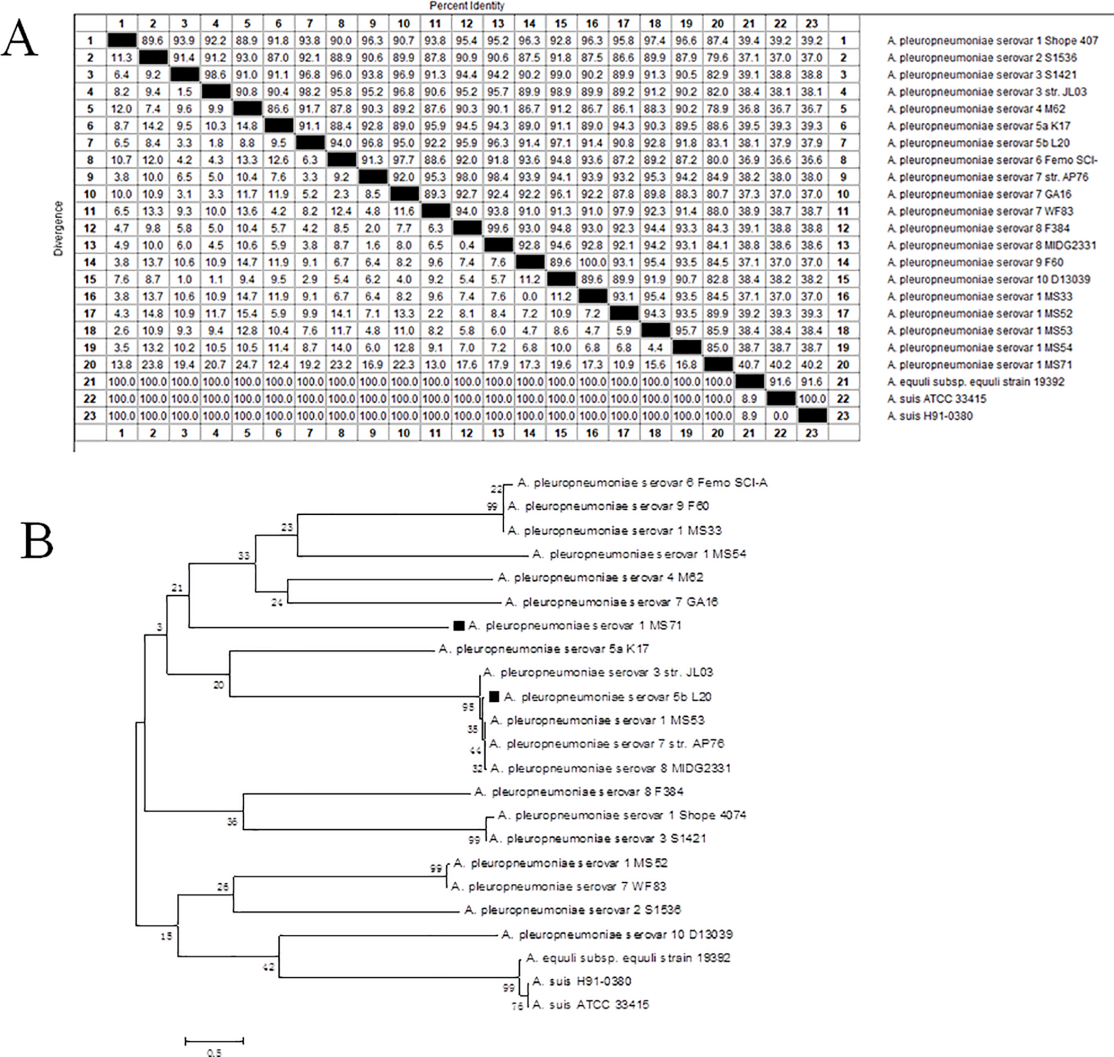
Percent identity (A) and Phylogenetic analysis (B) of *gal*T gene sequences from various strains. *Gal*T gene sequences from different strains were obtained from sequencing or downloaded from Genbank. DNASTAR, ClustalX1.83 and MEGA5.05 software were used to analyze sequences.

Multiple sequence alignment of *gal*T sequences were performed among APP isolated strains and reference strains using MegAlign software. *Gal*T sequences of 3 non-APP strains in *Actinobacillus*, A. suis strain ATCC33415, and H91-0380 and *A*. *equuli* subsp. *equuli* strain 19392 were included in the Genebank search. (Fig 2A). The *gal*T of L20 shared 83.1% identity with MS71. In APP strains, the identity between each two strains ranges from 78.9% (between M62 and MS71) to 100% (between F60 and MS33). Among APP strains and 3 other strains in *Actinobacillus*, the identities of *gal*T range from 36.6% to 40.7%. In *A*. *suis* and *A*. *equuli*, the identities of *galT* range from 91.6% to 100%. In short, *gal*T is highly conserved among APP strains.

Phylogenetic analyses of APP *gal*T sequences were performed among related sequences using ClustalX1.83 and MEGA 5.05 software (Fig 2B). APP serovar 1 clustered widely with different APP serotypes and in different phylogenetic trees. APP serovar 1 isolated strains from China widely distributed in different branches in the phylogenetic tree. Meanwhile, APP serovar 1 isolated strains and APP serovar 1 reference strain Shope 4074 didn’t in the same branch. Strains isolated in China like MS33, GA16, MS71, JL03 are widely distributed in different branches. *A*. *suis* and *A*. *equuli* are in the same branch, next to APP serovar 10 strain D13039.

### GALT was expressed and purified successfully in vitro

**Fig 3:**
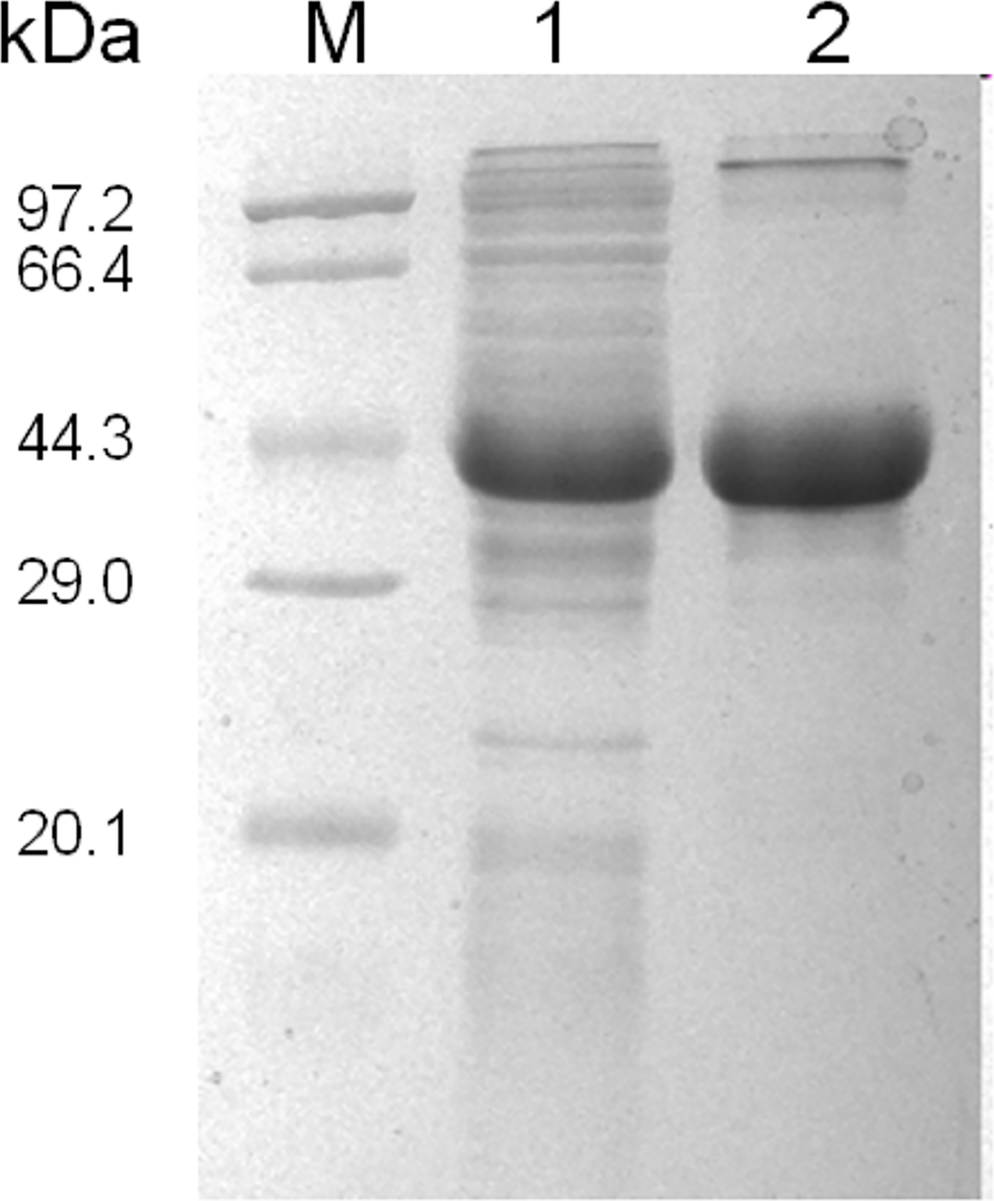
Expression and purification of His-tagged GALT protein; Lane M, protein marker; GALT recombinant strain induced by IPTG, his-tagged GALT protein (lane 1, whole cell lysates) and GALT purified by Ni-affinity chromatography (lane 2).

His-tagged GALT protein was induced with IPTG and was expressed in E. coli BL21 (Fig 3). Recombinant GALT protein was purified successfully by affinity chromatography and the size of this protein was about 40 Kilodalton (kDa) (Fig 3).

### GALT specific IgG was not detected in inactivated APP1 and APP5 immunization groups

**Fig 4:**
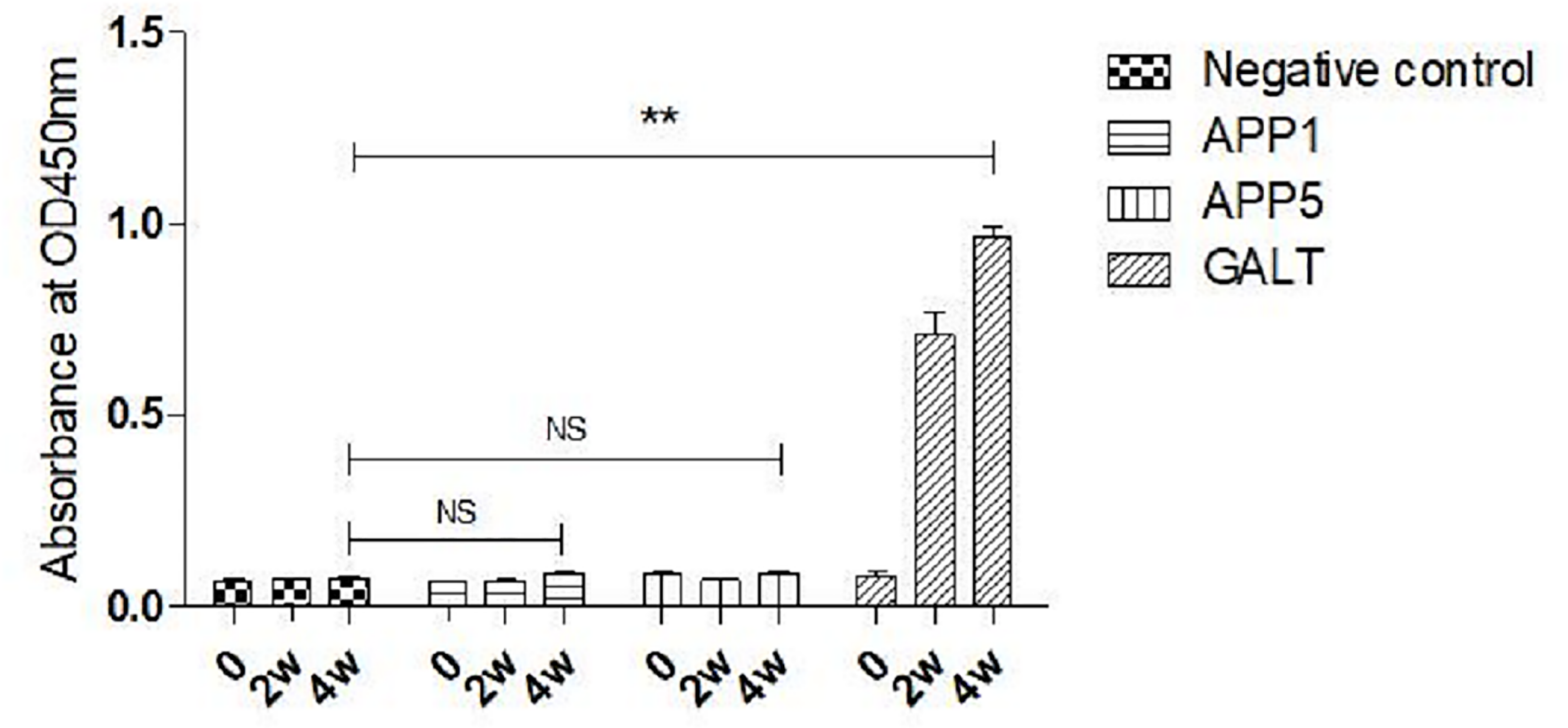
Detection of serum IgG from APP1, APP5, GALT vaccination and control groups by ELISA. Ninety-six well plates were coated with 200 ng/100 μl (per well) of purified GALT recombinant protein. The absorbance of each well was read at a wavelength of 450 nm using an ELISA reader (Bio-Rad, USA).

Animals were immunized with L20 and MS71 inactive whole cells, recombinant protein GALT and PBS as a negative control. Animals in different groups were immunized twice with a two-week interval and serum before immunization, 2 weeks and 4 weeks post initial vaccination. Serum samples were collected for IgG detection. 96-well plates for GALT IgG detection were coated with recombinant protein GALT. Serum in different groups was tested for the presence of IgG by indirect ELISA. In the negative control group, GALT specific IgG was not detected at 0, 2 and 4 weeks (Fig 4). In recombinant protein GALT group, the level of GALT specific IgG was elevated both after initial and booster immunizations. In APP1 and APP5 immunization groups, there was no GALT specific IgG detected (Fig 4).

### Partial immune protection was provided against APP serovar 1 and 5b

Animals were immunized with recombinant protein GALT in an APP challenge test. Two weeks post booster immunization, animals were challenged with lethal dose APP strong pathogenic strains MS71 and L20. The recombinant protein GALT was derived from L20 and animals were protected (75%, 6/8) against L20 challenge post immunization with recombinant protein GALT(Table 3). Animals in the all the negative control groups died acutely after challenge (Table 3). Although GALT was not derived from MS71, partial protection was provided from GALT when challenged with this strain (50%, 4/8)(Table 3). GALT also showed ability to protect against challenge with both virulent serovar 1 and 5b.

### GALT vaccinated animals showed less severe pathological signs in lung tissues

**Fig 5:**
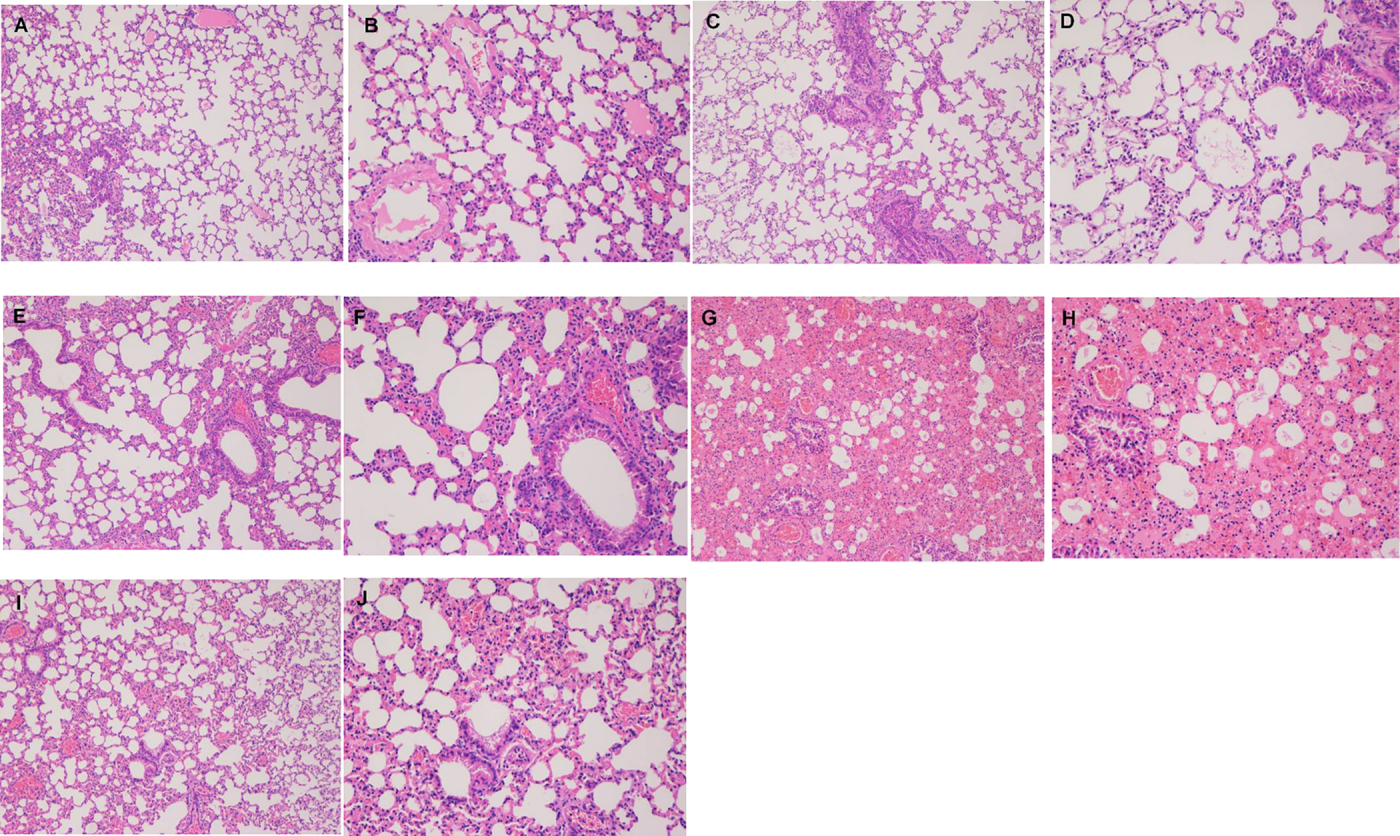
Histopathological examination of lung sections stained by HE. A (100×)& B (200×), normal control lung (without challenge). Group 1, c(100×)&d(200×), survival post challenged with APP 5b (immunized with GalT); group 2, e(100×)&f (200×), survival post challenge with APP 1 (immunized with GalT); group 3, g (100×)&h (200×), animals challenged with APP5b (PBS negative control); group 4, I (100×)&J (200×), animals challenged with APP 1 (PBS negative control).

Histopathologic examination was used to analyze the protective effects of GALT immunization. After MS71 or L20 challenge, lung tissue of mice in negative control groups underwent severe pathological changes. Compared with normal control group (Fig 5A and 5B), structures of pulmonary alveoli of lung tissue from challenged mice in negative groups were damaged, with heavy edematous lesions observed in the lung parenchyma (Fig 5G, 5H, 5I and 5J). Additionally, the lung tissue of negative control animals showed inflammation with infiltration of more neutrophils than that of normal control group. Finally, the tissues of surviving animals in GALT immune group had only moderate inflammation with infiltration of mixed mononuclear cells and neutrophils (Fig 5C, 5D, 5E and 5F).

### Infiltration of neutrophils are reduced in GALT immune group

**Fig 6:**
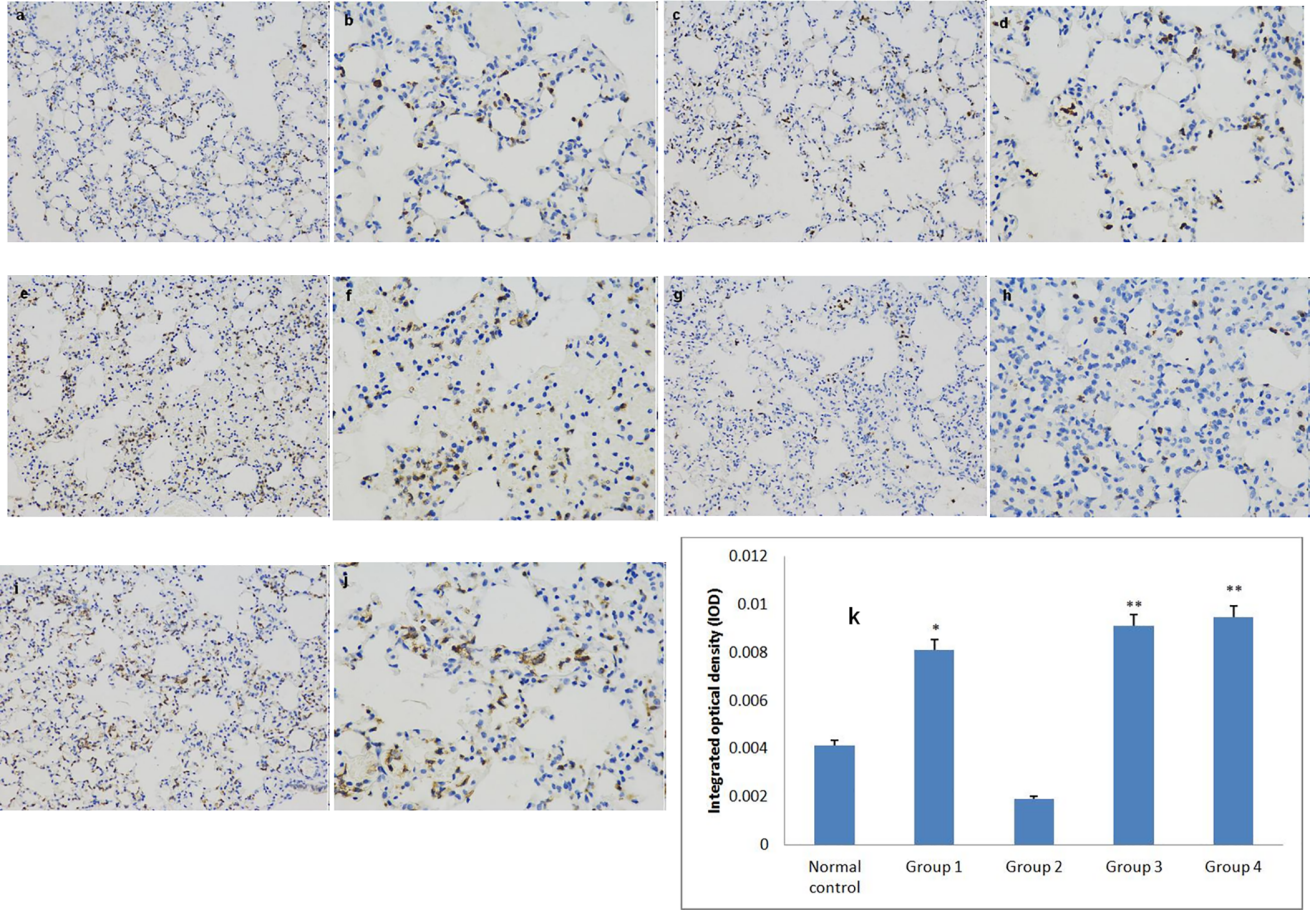
Immunohistochemical analysis of neutrophilic infiltration. Mice of each group were sacrificed post-challenge and lungs were collected for histopathology (a-j). Integrated optical density (IOD) of normal group served as control and groups with significant differences were noted as asterisks. Myeloperoxidase (Mpo) is a specific antigen for neutrophils detection, which is used in IHC. P-values of<0.05 were considered as statistically different and were represented with one asterisk. P-values of<0.001 were represented with two asterisks. A (100×)& b (200×), normal control. Group 1, c(100×)&d(200×), survival post challenge with APP 5b (immunized with GalT); group 2, e(100×)&f (200×), survival post challenge with APP 1 (immunized with GalT); group 3, g (100×)&h (200×), animals challenged with APP5b (PBS, negative control); group 4, I (100×)&J (200×), animals challenged with APP 1 (PBS, negative control); k, IOD in different group.

Susceptible/untreated mice showed severe clinical signs (labored respiration) after APP challenge was administered. Those showing clinical signs indicating APP infection were euthanized immediately; mice showing no, or mild, clinical signs were kept alive until the end of the experimental trial Macroscopic observation of lungs from negative control mice showed severe lung lesions with edema, hemorrhage and cyanosis. Lungs from surviving mice in the GALT immunization group were similar to those from the normal control. After challenge with APP L20 or MS71, the infiltration of neutrophils in the lung tissues in these two groups was significantly greater than that in normal controls (Fig 6). Mean Density of neutrophilic infiltration was much higher in the MS71 challenged negative controls than in the normal control(P<0.001)(Fig 6). In the APP L20 and MS71 challenged GALT immune groups, the infiltration of neutrophils in the lung tissues of surviving animals are less than that in negative control (Fig 6). Compared with the Mean Density of negative control, those of GALT immune groups displayed significantly reduced neutrophil infiltration (Fig 6). The results of IHC analysis of macrophages indicate that there is no significant difference between normal group and challenged groups (S2 File).

### Anti-GALT serum mediate neutrophils kill function

**Fig 7:**
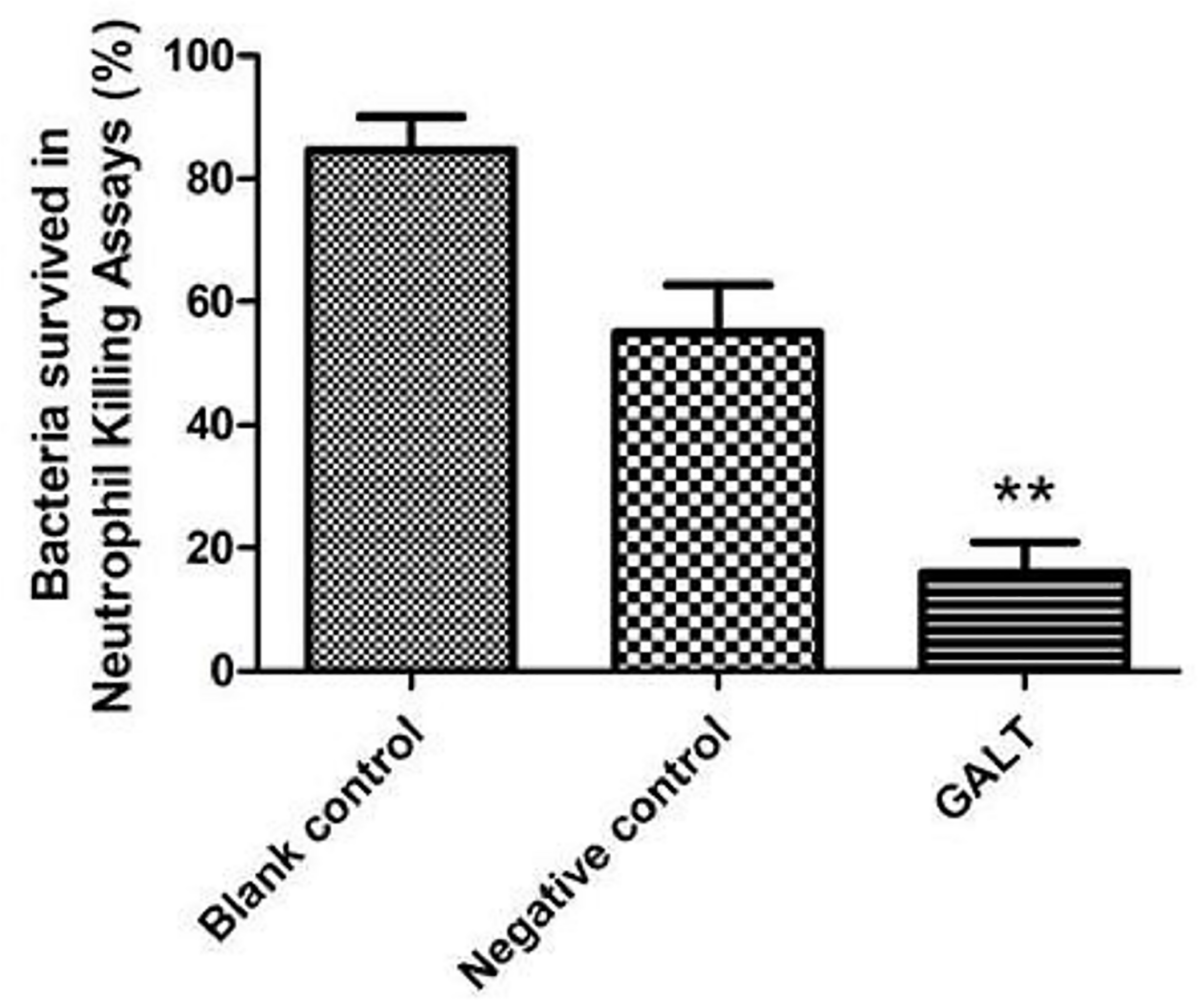
Neutrophil induced killing assay. Bacterial killing by murine neutrophils under opsonizing conditions. The percentage of bacteria survived in opsonophagocytic killing assay was calculated as (CFU with neutrophils/CFU without neutrophils) × 100%.

Survival rate of APP L20 incubated with anti-GALT antibodies was significantly reduced compared to that with the negative control serum (PBS immune serum) (P<0.001, Fig 7). Compared with survival rates of blank and negative control, survival rate of GALT group was reduced to 20%. This indicates that the oposonization of phagocytosis of neutrophils could be efficiently induced by anti- GALT antibodies.

## Discussion

APP, as one of the most important bacterial pathogens of swine, has been studied for several decades. APP causes a severe respiratory system disease of swine with high mortality rates, and incurs significant economic losses in the swine agronomy worldwide. Antibacterial agents have been viewed as the first choice to treat the diseases caused by bacteria. However, the reported abuse and overuse of antibacterial agents is selecting for an ever-growing population of resistant pathogens, including APP [41–44]. The use of vaccines has proven to be a better choice in efforts to control PCP outbreaks. Apart from use of traditional inactivated vaccine, many studies have focused on new vaccines to control PCP [45–52]. GALT was demonstrated as an efficient antigen against APP, and could be taken as a potential vaccine candidate [36]. A different APP serovar 1 isolated strain MS71 was used in this study to analyze the cross-protection effects of recombinant protein GALT. In present study, it was demonstrated recombinant GALT protein of APP serovar 5b strain L20 was cross protective against APP serovar 1 strain MS71 challenge.

Bioinformatics analysis shows that *gal*T gene is highly conserved and widely distributed in different APP strains, making it an promising candidate as a basis for a cross protective vaccine. The results of phylogenetic analysis demonstrate that almost all APP strains are distributed in one phylogenetic branch. L20 and MS71, however are classified in a small side branch. The similarities between each two APP strains in this study range from 78.9% to 100% and that between L20 and MS71 is 83.1%. It is reported that *Plp*B across different *P*. *multocidas* strains exhibited a high level of homology (80.8%-99.4%), and the mice anti-rPlpB sera was cross-reactive with several *P*. *multocida* strains [53]. Outer membrane protein F (*Omp*F) gene of *Escherichia coli* strain shares an identity (90–100%) with other select *E*. *coli* (46.7%) and *Shigella* (52.8%) strains. Immunological tests show that the recombinant OmpF induces an effective immune response and provides a protection against multi-strain challenges [54]. The outer membrane protein, LamB, of *Vibrio alginolyticus* was also demonstrated as an effective cross protective antigen, which showed cross-reactivity [55]. ApfA, VacJ and PalA of APP were also studied for their potential as vaccine candidates. However, all of these antigens could induce antigen specific antibodies, but failed to induce protection against challenge [56, 57]. In the present study, recombinant GALT protein derived from L20 provided partially protection against MS71 challenge. The protection qualities of GALT may also exist between serovar 5b and the other serovars.

GALT could induce a high level antibody response, indicating that this antigen has high immunogenic properties. Immunization of animals with inactivated whole-cell APP MS71 or L20 does not elicit any GALT specific antibodies. Therefore, GALT may be of use as a diagnostic antigen to differentiate animals infected or exposed to APP from those that were vaccinated against this agent (‘infection’ sera vs. ‘vaccine’ sera).

In this study, the results of IHC analysis demonstrated that infiltration of neutrophils in negative control (challenged) group was significant higher than in the normal control at the early stages of infection. Meanwhile, in the immunization group, infiltration of neutrophils is lower than that in the negative control. The lesions caused by APP are characterized by necrosis, hemorrhage and neutrophil infiltration in the early stages, followed by vascular thrombosis, edema and macrophage infiltration with fibrinous pleurisy[58]. In an acute inflammatory response, neutrophils are important effector cells that serve to clear extracellular pathogens[59]. Neutrophils are the predominant inflammatory cells infiltrating the alveolar spaces during early APP infection [60].

Anti-GALT serum induces phagocytosis of neutrophils thereby stimulating an innate immune response in the process of APP infection (Fig 7). Neutrophils are the major cell type in an innate immune response, which acts as primary line of defense against invading microbial pathogens[61–63]. As a professional phagocyte, the function of neutrophils is engulfment, killing and clearance of bacteria [64]. In addition, neutrophils are involved in the activation, regulation and effector functions of innate and adaptive immune cells [59].

We demonstrated that GALT provided immune protection in the murine model, and confers partial cross protection against heterologous APP sevorars. *Gal*T is a highly conserved gene which is distributed throughout 10 reference strains as well as isolated strains used in this study. The sequences of *gal*T are conserved and the identities between any of the APP strains analyzed range from 79% to 100%. GALT specific antibodies could not to be detected in immune sera of animals vaccinated with inactivated APP MS71 or L20. Animals in the negative control group died rapidly after challenge, while the immunization groups provided partial protection. This demonstrated that immunization of recombinant GALT from APPsevorar 5b strain L20 confers cross protection against heterologous APP serovars. Histopathological and IHC analysis demonstrated that tissue damage was alleviated and the infiltration of neuthophils in the animal lung tissue was reduced post immunization with the application of GALT vaccine. In addition, bacterial phagocytosis of neutrophils was found to be significantly promoted by GALT specific antibodies. The present study demonstrated the function of GALT protein in a murine model, which is an important initial step in vaccine preparation trials. In the future, the potential vaccine candidate used in the present study should be tested for protective efficacy in pigs.

## Supporting information

S1 File Nucleotide sequences used in this study. (PDF)

S2 File Figure A. Immunohistochemical analysis of macrophages.
(PDF)
