# Supplementary material for: Galactose-1-phosphate uridyltransferase (GalT), an in vivo-induced antigen of Actinobacillus pleuropneumoniae serovar 5b strain L20, provided immunoprotection against serovar 1 strain MS71
Source: PLoS One. 2018 Jun 1;13(6):e0198207. doi: 10.1371/journal.pone.0198207 (PMC5983418; doi:10.1371/journal.pone.0198207)
Supplement: S1 File — (PDF) [file pone.0198207.s001.pdf]

GenBank accession numbers for nucleotide sequences in this study:

GenBank MG599494-MG599511:

BankIt2067381	Shope4070	MG599494
BankIt2067381	S1536	MG599495
BankIt2067381	S1421	MG599496
BankIt2067381	M62	MG599497
BankIt2067381	K17	MG599498
BankIt2067381	Femo	MG599499
BankIt2067381	WF83	MG599500
BankIt2067381	F384	MG599501
BankIt2067381	F60	MG599502
BankIt2067381	D13039	MG599503
BankIt2067381	MS71	MG599505
BankIt2067381	GA16	MG599506
BankIt2067381	MS52	MG599508
BankIt2067381	MS53	MG599509
BankIt2067381	MS54	MG599510
BankIt2067381	MS33	MG599511
