# Supplementary material for: Galactose-1-phosphate uridyltransferase (GalT), an in vivo-induced antigen of Actinobacillus pleuropneumoniae serovar 5b strain L20, provided immunoprotection against serovar 1 strain MS71
Source: PLoS One. 2018 Jun 1;13(6):e0198207. doi: 10.1371/journal.pone.0198207 (PMC5983418; doi:10.1371/journal.pone.0198207)
Supplement: S2 File — Figure A. Immunohistochemical analysis of macrophages. (PDF) [file pone.0198207.s002.pdf]

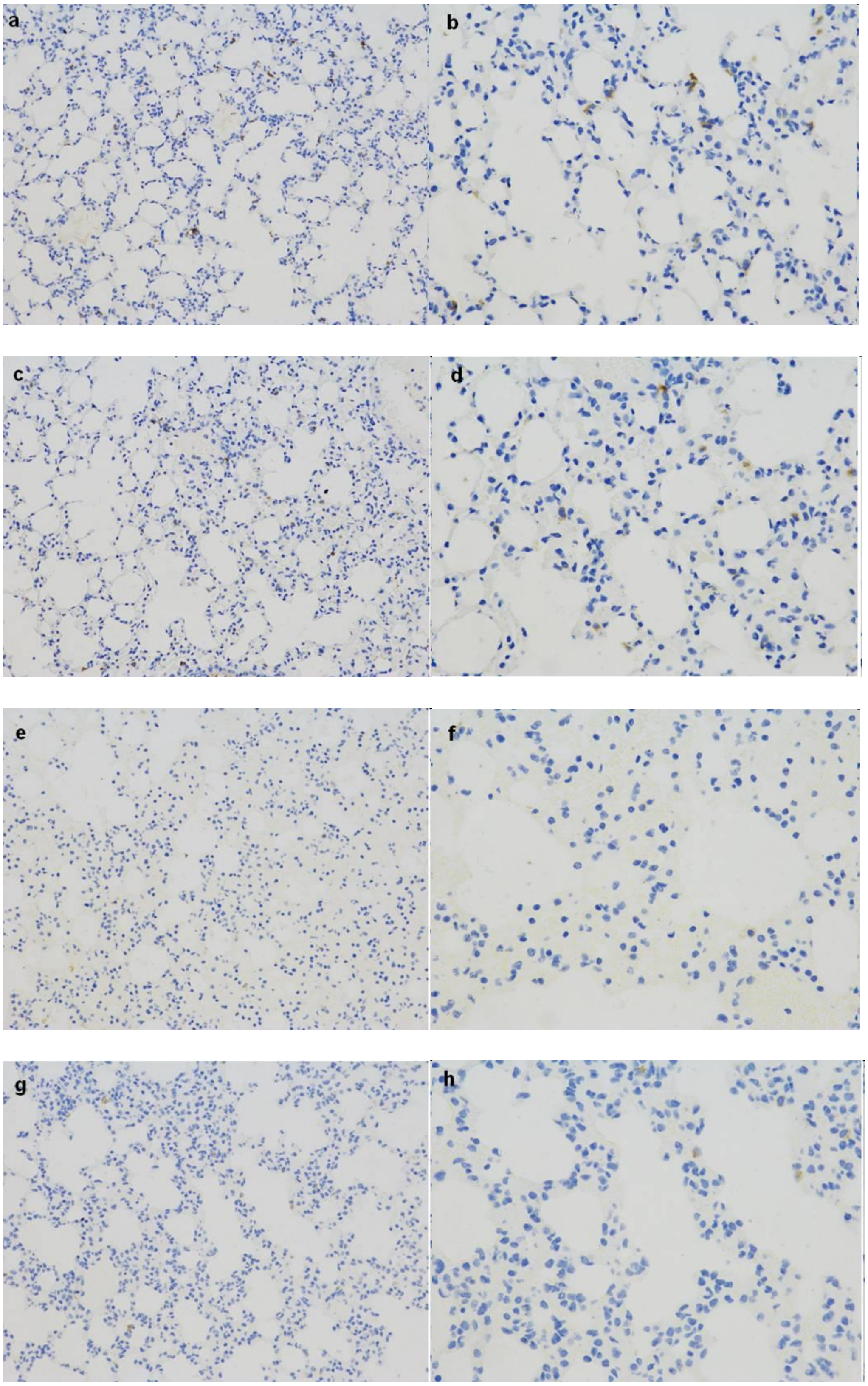
a
b
c
d
e
f
g
h

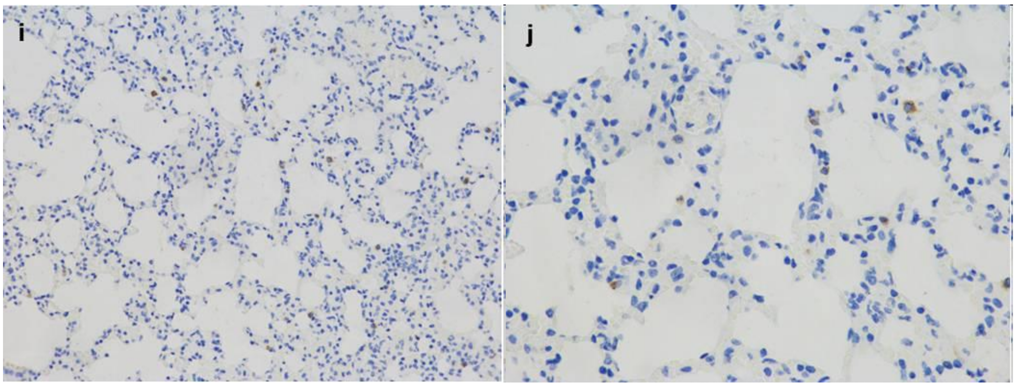

k

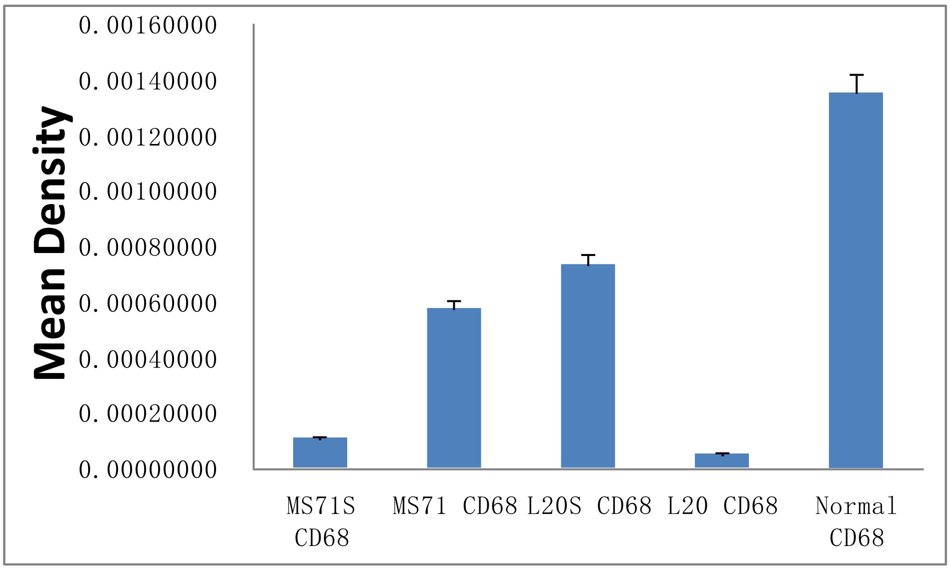

Figure A. Immunohistochemical analysis of macrophages. a (100×)& b (200×), normal control; c(100×)&d(200×), survival post challenged with L20(immunized with GalT); e(100×)&f(200×), animals challenged with L20(negative control); g(100×)&h(200×), survival post challenged with MS71(immunized with GalT); I(100×)&J(200×), animals challenged with MS71(negative control).
